# First Eocene lizard (Squamata) from Balkanatolia documents overwater dispersal of Paleogene pleurodontan iguanians

**DOI:** 10.1038/s41598-026-59284-2

**Published:** 2026-07-06

**Authors:** Georgios L. Georgalis, Krister T. Smith, Kevin D. Mulcahy, Aidan Dunlay, Johan Knoff, Faruk Ocakoğlu, Alexis Licht, Grégoire Métais, K. Christopher Beard

**Affiliations:** 1 Institute of Systematics and Evolution of Animals, Polish Academy of Sciences, Sławkowska 17, 31-016 Kraków, Poland; 2 Senckenberg – Leibniz Institution for Biodiversity and Earth System Research, Senckenberg Research Institute and Natural History Museum Frankfurt/M, Senckenberganlage 25, 60325 Frankfurt am Main, Germany; 3 Faculty of Biosciences, Institute for Ecology, Evolution, and Diversity, Goethe University Frankfurt, Max-von-Laue Strasse 9, 60325 Frankfurt am Main, Germany; 4 Department of Ecology and Evolutionary Biology, University of Kansas, Lawrence, KS USA; 5 Biodiversity Institute, University of Kansas, Lawrence, KS USA; 6 Department of Geological Engineering, Eskişehir Osmangazi University, Eskişehir, Turkey; 7 CNRS, IRD, INRAE, CEREGE, Aix Marseille University, Aix-en-Provence, France; 8 Centre de Recherche sur la Paléobiodiversité et les Paléoenvironnements, UMR 7207 (CNRS, MNHN, UPMC), Sorbonne Université, Muséum National d’Histoire Naturelle, Paris, France

**Keywords:** Iguania, Pleurodonta, Anatolia, Biogeography, Dispersals, Ecology, Ecology, Evolution, Solid Earth sciences, Zoology

## Abstract

We here describe a fossil jaw fragment from the middle Eocene (Lutetian) Uzunçarşıdere Formation in Anatolia, pertaining to a pleurodontan iguanian. This region during most of the Eocene was part of an isolated insular landmass, called Balkanatolia, that is mostly known for its peculiar and diverse insular mammalian fauna. The lizard specimen represents the first evidence of Eocene reptiles from Balkanatolia, providing a first glimpse into the herpetofaunas of this, now lost, insular landmass. The fossil material is rather fragmentary, precluding a more precise identification, but its distinctive tooth morphology confirms the presence of pleurodontan iguanians in the region for the first time. The presence of a distinct mesiodistal groove along the apical crest of the central cusp in the teeth of the Anatolian pleurodontan is of particular interest, as this feature is otherwise observed only in the Eocene genera *Cadurciguana* from Western Europe and *Parasauromalus* from North America, plus the extant American lineages of Dactyloidae, Leiocephalidae, and Polychrotidae. Interestingly, this feature seems to be absent from all other pleurodontans, including the abundant Eocene corytophanid *Geiseltaliellus*. The new find indicates another successful overseas dispersal of pleurodontans, a group that has repeatedly colonized distant landmasses, including oceanic islands, throughout its evolutionary history. Taking into consideration the diversity and abundance of pleurodontans in the Eocene of Europe and the total absence of the group from coeval Africa and Asia, it is likely that the new Anatolian iguana arrived from the former continent sometime between the early and early middle Eocene.

## Introduction

Lizards have a fascinating biogeographic history, that encompasses an array of dispersal events, often including long marine barriers, as aptly demonstrated by the extant and fossil distribution of various groups, which includes even remote oceanic islands. Indeed, several lizard groups, from tiny gekkotans to large Monitor Lizards are known to be capable of long distance, overwater, dispersals, usually facilitated via rafting on “floating islands of vegetation”, a fact ultimately enabling them to colonize distant and remote terrains (e.g.,^1–8^). Among the most successful lizard lineages regarding such overwater dispersals are pleurodontan iguanians (i.e., pleurodontans), which record not only the almost prosaic dispersals of anoles (*Anolis*) to and among Caribbean islands^9^ but also the longest suggested overwater dispersal among all terrestrial vertebrates (more than 8000 km), from North America to the Fiji Islands^1,8^.

Pleurodonta (also known, depending on the applying terminology, as Pan-Iguanidae or Iguanidae sensu lato or Iguanoidea or Neoiguania [see below for notes on the nomenclature of the group]) represent a very diverse group of lizards^10–14^. They are the sister group of Acrodonta (i.e., chameleons, agamas, spiny-tailed lizards, and their relatives), together comprising Iguania^10,15–17^. Extant Pleurodonta have a rather disjunct and scattered geographic distribution, which comprises the Americas (including Caribbean and Galapagos Islands), Madagascar, and a few islands in Oceania^5,7–10,12,18–20^. The fossil record implies an even broader distribution that also encompassed the Paleogene (and perhaps also the Late Cretaceous) of Europe and the Late Cretaceous of Asia (e.g., ^21–25^). The disjunct extant distribution of pleurodontans, coupled with their broad fossil record across other continents, bears witness to a fascinating and rather complex evolutionary and biogeographic history of vicariance and overseas dispersal events^1,5,7,8,23^.

Here we describe a fossil jaw fragment of a pleurodontan from the middle Eocene (Lutetian) of the Uzunçarşıdere Formation in Anatolia. This area, during the early and middle Eocene, was part of an isolated insular landmass, called Balkanatolia, that hosted a uniquely endemic mammal fauna^26–31^. The Balkanatolian mammal fauna includes clades showing biogeographic affinities with each of the adjacent mainland faunas of Europe, Asia, and Africa. Several of these mammal clades diversified after colonizing Balkanatolia, possibly in response to iterative episodes of partial inundation of that isolated landmass^26–30,32–35^. The new fossil specimen reported here represents the first reptile described from the insular fauna of Balkanatolia. It indicates another successful episode of overseas dispersal of pleurodontans during their evolutionary history. Biogeographic implications of the new find and the potential origin of the new pleurodontan are discussed.

### Institutional abbreviations

EOU-UCF, fossil specimens from the Uzunçarşıdere Formation at Eskişehir Osmangazi University, Eskişehir, Türkiye; ISEA, Institute of Systematics and Evolution of Animals, Polish Academy of Sciences, Kraków, Poland; MNHN, Muséum national d’Histoire naturelle, Paris, France; MZUSP, Museu de Zoologia da Universidade de São Paulo, São Paulo, Brazil; SMF, Herpetology collection, Senckenberg Research Institute and Natural History Museum, Frankfurt am Main, Germany; SMF-ME, Messel vertebrate collection, Senckenberg Research Institute and Natural History Museum, Frankfurt am Main, Germany; SMF-PH, Paleoherpetology collection, Senckenberg Research Institute and Natural History Museum, Frankfurt am Main, Germany; UM, Institut des Sciences de l’Évolution, Université de Montpellier, Montpellier, France (formerly abbreviated as “USTL”).

## Material and methods

The fossil material presented herein is permanently curated at the collections of Eskişehir Osmangazi University (Eskişehir, Türkiye). The material was collected from the AK-1 site of the Lülük Member of the Uzunçarşıdere Formation in the Orhaniye Basin of north-central Türkiye. No permits were required for the present study, which complied with all relevant regulations. For comparative purposes, multiple extant and extinct pleurodontan skeletons were studied at the collections of ISEA, MNHN, SMF-ME, SMF-PH, and UM. Scanning Electron Microscope (SEM) images of the fossil material were taken at the Microscopy and Analytical Imaging Research Resource Core Lab, University of Kansas, using a Hitachi S4700 Cold-Field Emission Scanning Electron Microscope.

### Geological setting

**Fig. 1:**
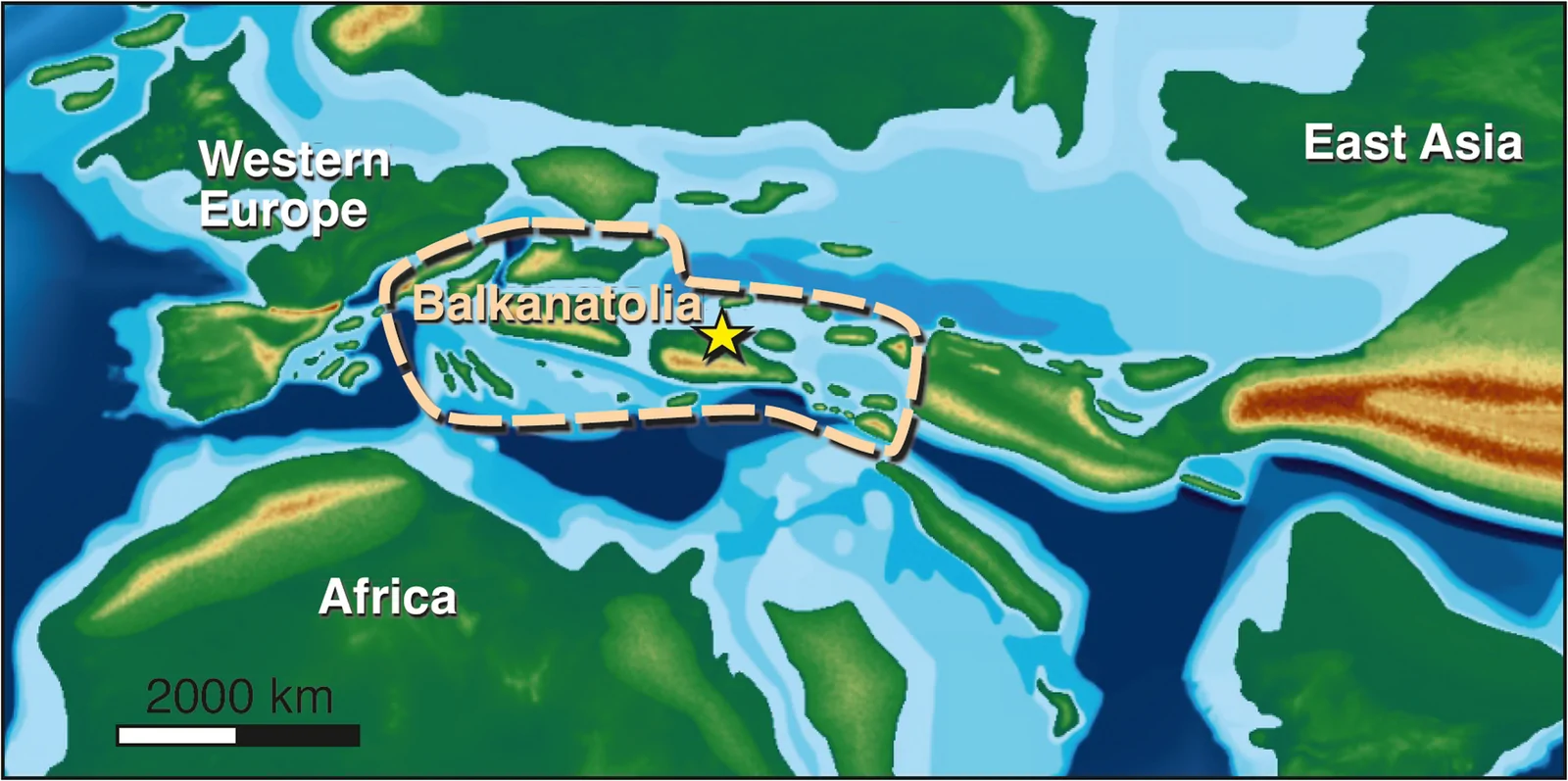
Map of the western Neotethys during the latest Lutetian, at the time of the Uzunçarşıdere Formation, modified from Montheil et al. (2026). Star indicates the position of the Uzunçarşıdere Formation.

The fossil material was collected from the Lülük Member of the Uzunçarşıdere Formation in the Orhaniye Basin of north-central Türkiye. The site that yielded the new lizard specimen is called AK-1. It is located near the village of Sarıbeyler in the Orhaniye Basin. Precise locality coordinates are maintained at the Division of Vertebrate Paleontology, University of Kansas Biodiversity Institute. These strata have been dated to the Lutetian (middle Eocene), approximately 44–43 Ma on the basis of magneto-stratigraphy and detrital zircon geochronology^31,36^. This rock unit is well known for yielding a highly endemic mammalian fauna that includes anatoliadelphyid and herpetotheriid metatherians, pleuraspidotheriid “condylarths,” palaeoamasid embrithopods, palaeochiropterygid bats, and omomyid primates, among other taxa^26,28,29,32,33,35,37,38^. During the early Paleogene, the Orhaniye Basin and broader Anatolia were part of a semi-continuous strip of land located along the Eurasian Neotethyan margin, between Western Europe and the Middle East, known as Balkanatolia^30,39^ (see Fig. 1). Balkanatolia acted as an independent biogeographic province during the early and middle Eocene, and possibly during earlier times of the Paleogene^26,27,29–31^. During the Paleocene and most of the Eocene, Balkanatolia was separated from other landmasses by narrow seaways and experienced numerous episodes of partial inundation^30,40^, which favored the isolation of the regional fauna (Fig. 1). Balkanatolia fully emerged as a continuous landmass connected to Eurasia during the Bartonian, 40 to 38 million years ago; this geographical continuity paved the way for the arrival of Asia-derived mammals and the end of Balkanatolian endemism^30,34,39^.

### Nomenclatural note on the usage of the name Pleurodonta

The traditional family Iguanidae (in the Linnean system) was critically examined by Frost & Etheridge^10^, who found no morphological evidence for the monophyly of the group (although they did not demonstrate paraphyly either). Hence, because (1) they insisted on monophyly in higher taxonomy, (2) the ICZN^41^ governs nomenclature only up to the family level, and (3) “iguanid” monophyly was in doubt, Frost & Etheridge^10^ elected to break up that taxon into eight families, each of which appeared to be monophyletic: Corytophanidae, Crotaphytidae, Hoplocercidae, Iguanidae, Opluridae, Tropiduridae, Phrynosomatidae, and Polychrotidae. They recognized the higher taxon Pleurodonta as encompassing these families (Pleurodonta, of course, was just as ambiguous as the traditional Iguanidae, but it was not formally speaking a “family”). Soon thereafter, molecular evidence for a monophyletic Iguanidae of traditional composition was presented^42^. Nevertheless, Frost et al.^12^ doubled down on their taxonomic changes, citing “acceptance” of their taxonomy in the intervening years (and admittedly the general approach of elevating sub-familial ranks to family level has been extremely widely practiced in herpetology since then, even when the families that so arise have a single genus in them); their molecular analyses furthermore cast doubt on the monophyly of some of these new families, which led Frost et al.^12^ to recognize a number of new family-level taxa: Leiocephalidae, Leiosauridae, Liolaemidae, Dactyloidae. These nomenclatural changes have been widely accepted^7,8,13,15,43–46^. More recently, Vidal and Hedges^47^ resurrected the name Iguanoidea to encompass all pleurodontan iguanians (see also^43^), while de Queiroz et al.^48^, applying the PhyloCode system, insisted on *Iguanidae*, for which Smith^16,17^ defined the clade name *Pan-Iguanidae*. In any case, the name Pleurodonta has been widely accepted in recent herpetological (e.g., ^7,44–46,49^) and palaeoherpetological (e.g., ^13,50–55^) literature, and we follow this practice here.

### Systematic palaeontology

Squamata Oppel, 1811^56^

Iguania Cuvier, 1817^57^

Pleurodonta Cope, 1864^58^ (sensu^12^)

Pleurodonta indet.

Fig. 2

**Fig. 2:**
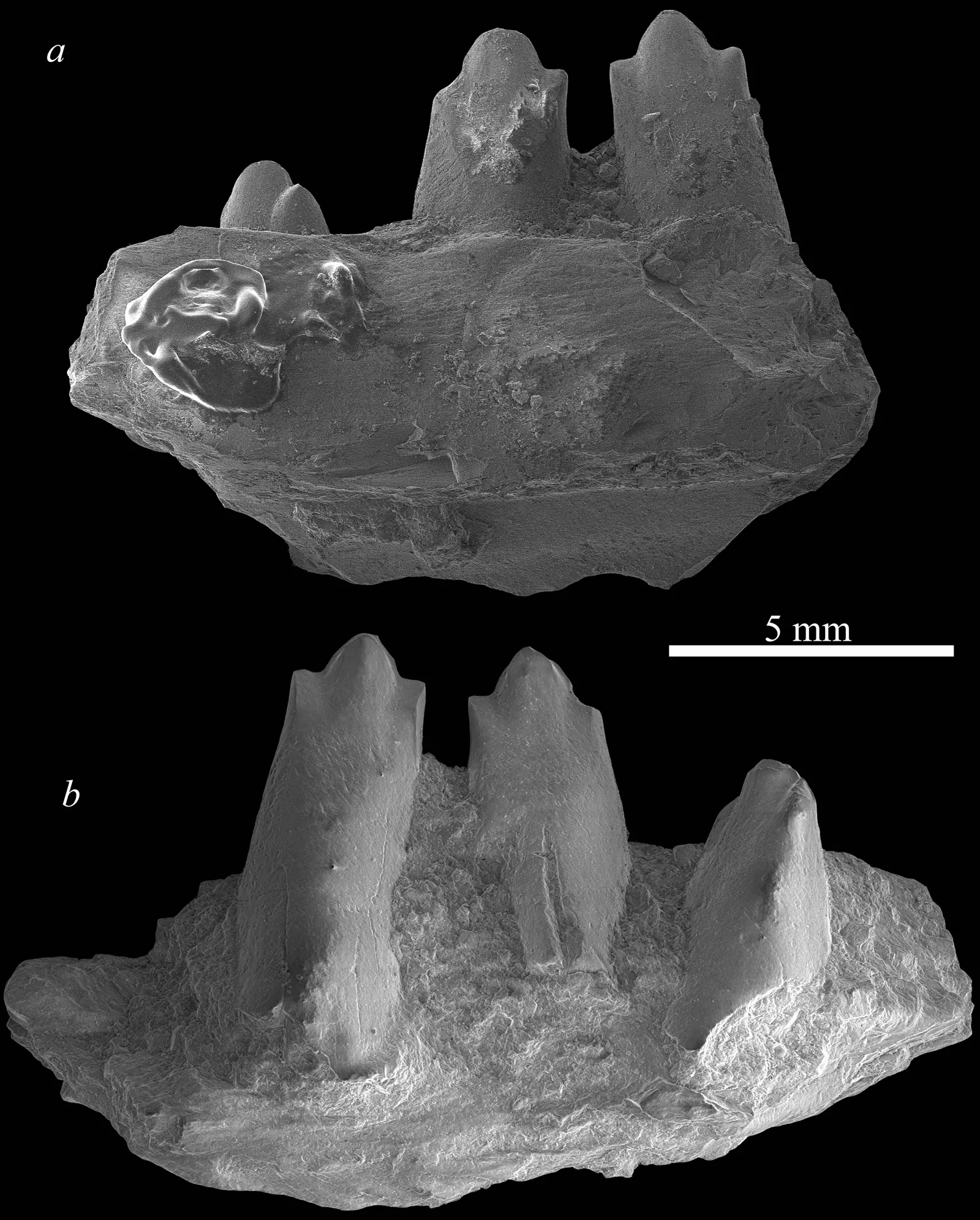
Pleurodonta indet. from the Uzunçarşıdere Formation. SEM image of fragment of jaw (EOC-UCF-15) in labial (**a**) and medial (**b**) views. SEM image made by K.D.M.

***Material***. A fragment of a jaw (EOC-UCF-15).

***Description***. The jaw fragment EOC-UCF-15 is highly incomplete, preserving only three teeth and one empty tooth position, plus part of the dental shelf (Fig. 2). It is unclear whether it represents part of a dentary or a maxilla. The dentition is pleurodont. Among the three preserved teeth, two are fully preserved and the third one is rather damaged and incomplete. The teeth are distinctly tricuspid; they possess a prominent central cusp and two weaker accessory cusps that are separated by apicobasal grooves from the central cusp both lingually and labially^59^. A prominent, mesiodistal groove runs along the apical edge of the central cusp, dividing it into lingual and labial portions. The teeth are cylindrical and robust, with a relatively wide base. There is a slight inclination in the two well preserved teeth.

## Results

### Taxonomic attribution

The jaw fragment EOC-UCF-15 is most similar to those of pleurodontans based on the pleurodont dentition and the distinctly tricuspid teeth with a distinct central and two weaker accessory cusps^18,23,59^. It is, however, possible that these characteristics are plesiomorphic for Iguania, in which case they do not provide any evidence for a relationship with Pleurodonta specifically^10,23^. Furthermore, tricuspid teeth can occur in some Lacertoidea, namely certain members of teiids, lacertids, and gymnophthalmids (e.g., ^60–63^). In lacertids and gymnophthalmids though the mesial accessory cusp usually appears before the distal accessory cusp, unlike in Pleurodonta, where the reverse is true. Τeiids have the base of the tooth often swollen and embedded inside a deposit of cementum (see^64^); an apical groove is absent in all teiids we had available for study (*Crocodilurus lacertinus* MZUSP 92090; *Tupinambis quadrilineatus* MZUSP 84921; *Tupinambis teguixin* ISEA R/32, ISEA R/33, ISEA R/37, ISEA R/440, ISEA R/441, ISEA R/442, *Salvator rufescens* ISEA R/260, *Ameivula ocellifera* MZUSP 22341; *Ameiva ameiva* MZUSP 43047 [KTS, pers. obs.])—moreover, the posterior tricuspid teeth of teiids are also anteroposteriorly expanded (and not labiolingually), so they have a different form. In gymnophthalmids also, teeth of certain members of this group can possess distinct tricuspidity with lateral cusps much smaller than the medial one, however, this tricuspidity is confined to the posterior larger teeth and not to the anterior(most) ones, which are smaller and conical, and is overall different than the tricuspidity observed in pleurodontans (e.g.,^61,63^). Tricuspidity can also appear in other, distantly related groups, e.g., species of the scincid genus *Chioninia* (with one species even approaching extreme multicuspid morphology; see^65^) or some Cretaceous polyglyphanodontians (e.g.,^66^), but they still differ from pleurodontans in the shape and size of the accessory cusps. As a further note, it has been suggested that certain members of Scincoidea (e.g., xantusiids) can also possess somehow tricuspid teeth, however, that “tricuspidity” (if this should be indeed termed as such) is usually faintly expressed, and the teeth do not have a central cusp separated from the accessory cusps by both labial and lingual grooves (e.g., ^60^). Some characteristics of the tooth-bearing elements—a separation of the subnarial arterial and anterior superior alveolar foramina^23^ and a wrapping of the splenial underneath the subdental shelf^23,24^ might be synapomorphies that could help identify such incomplete remains, but the present Anatolian material is too fragmentary even for that. At the same time though, the alternatives are less plausible: Gymnophthalmidae and Teiidae are exclusively American (but see^67^ for plausible Eocene occurrences of the latter from France); tricuspid Eocene Lacertidae are very rare, with tricuspidity being not prominent anyway (with accessory cusps being very small; e.g., ^68,69^), while more lacertid taxa with even more “distinct” tricuspidity appear in the Oligocene (e.g., ^70–74^).

**Fig. 3:**
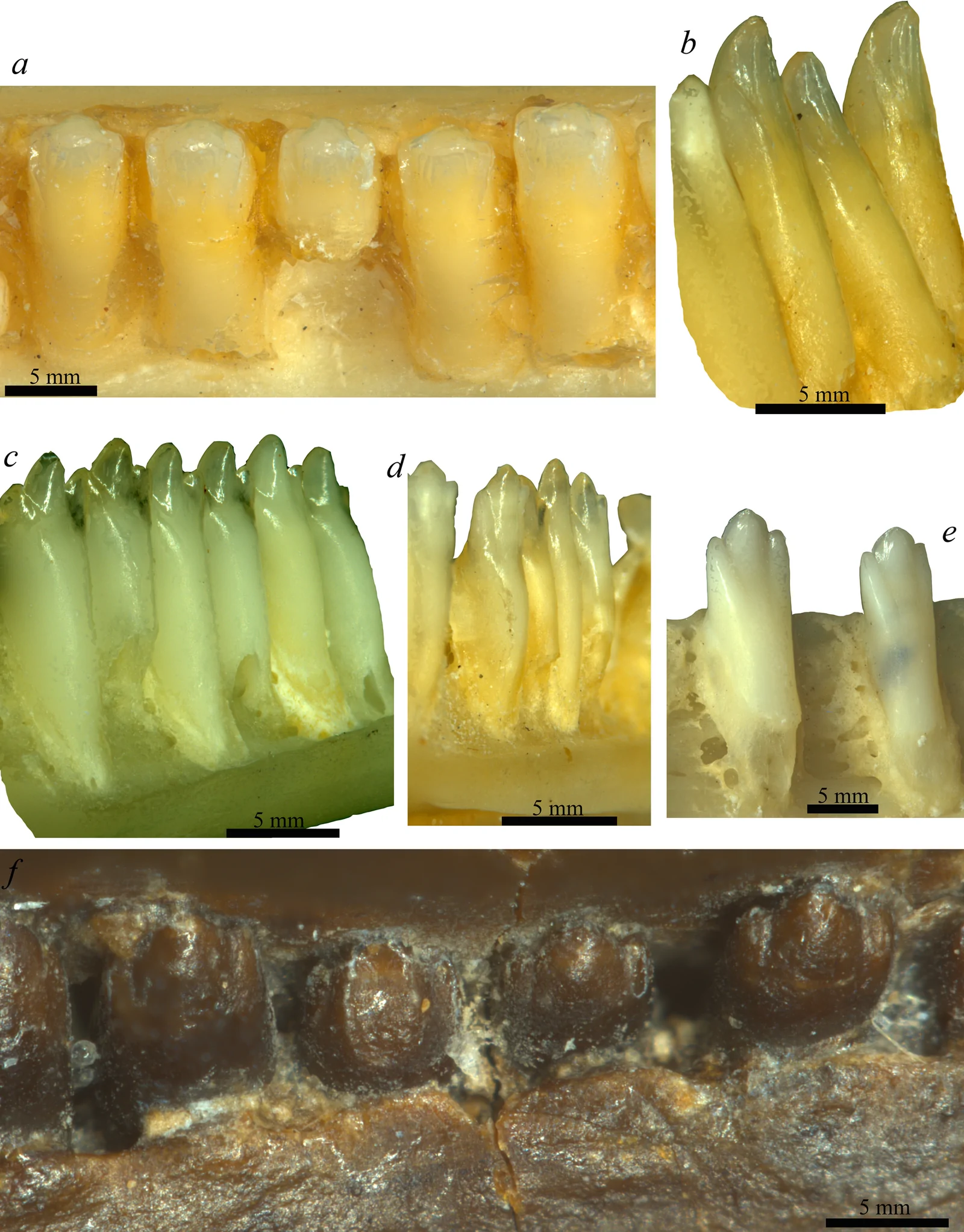
Close up of teeth of selected extant pleurodontans and the Eocene *Geiseltaliellus maarius* from Messel, showing the presence (**a–c**) or absence (**d–f**) of the mesiodistal groove along the apical crest of the central cusp of the teeth. **a–b**) *Polychrus acutirostris* (left dentary SMF 24870) in dorsal (**a**) and posteromedial (**b**) views; **c**) *Leiocephalus carinatus* (left dentary SMF-PH 308) in posteromedial view; **d**) *Enyalioides oshaughnessyi* (left dentary SMF 67590) in posteromedial view; **e**) *Corytophanes cristatus* (left dentary SMF-PH 291) in medial view; **f**) *Geiseltaliellus maarius* (right maxilla of paratype SMF-ME 1769) in dorsal view. All photographs made by K.T.S.

Among extinct genera of Pleurodonta from Europe, EOC-UCF-15 shares the highest resemblance with *Cadurciguana* Augé^75^, from the middle–late Eocene of France, particularly in terms of the robust and cylindrical teeth^55,62,75^. The mesiodistal groove along the apical crest of the central cusp in EOC-UCF-15 is also present in *Cadurciguana* (uniquely among European pleurodontans) and, notably, the North American *Parasauromalus* sp. (see^76^: fig. 8C), which Smith and Gauthier^76^ considered a stem relative of the extant *Polychrus*. Amongst extant taxa this groove is present in all examined Dactyloidae, Leiocephalidae, and Polychrotidae (*Anolis barbatus* SMF 79164, *Anolis carolinensis* SMF-PH 593, *Anolis conspersus* SMF-PH 317, *Anolis cuvieri* SMF 60830, *Anolis garmani* SMF-PH 89, *Anolis princeps* SMF 66729, *Anolis richardii* SMF 65821, *Anolis sagrei* SMF-PH 386, *Anolis serranoi* SMF 78838, *Leiocephalus carinatus* SMF-PH 308 (Fig. 3c), and *Polychrus acutirostris* SMF 24870 (Fig. 3a–b)) but is absent in all examined members of other pleurodontan clades, viz. Corytophanidae, Crotaphytidae, Hoplocercidae, Iguanidae, Opluridae, Phrynosomatidae, and Tropiduridae (KTS and GLG, pers. obs.; *Basiliscus plumifrons* SMF-PH 595, *Basiliscus vittatus* SMF-PH 853, *Chalarodon madagascariensis* SMF 71025, *Corytophanes cristatus* SMF-PH 291 (Fig. 3e), *Crotaphytus* ?*antiquus* SMF-PH 286, *Dipsosaurus dorsalis* SMF-PH 296, *Enyalioides oshaughnessyi* SMF 67590 (Fig. 3d), *Gambelia* sp. SMF-PH 414, *Laemanctus longipes* SMF-PH 292, *Laemanctus serratus* SMF-PH 294, *Oplurus cyclurus* SMF 79191, *Petrosaurus thalassinus* SMF-PH 92, *Phymaturus* sp. SMF-PH 875, *Sauromalus ater* SMF-PH 1025, *Sceloporus* sp. SMF-PH 849). Sometimes a groove is found in the latter taxa on the lingual side of the mesial and distal carinae, but it does not extend apically over the central cusp. The apical groove is absent, to our knowledge, in the other lizard clades with tricuspid members (e.g., Lacertoidea). Thus, this apical groove provides the strongest evidence that EOC-UCF-15 is not just a pleurodontan iguanian but specifically could pertain to the total clades of *Anolis*, *Leiocephalus*, or *Polychrus* (Dactyloidae, Leiocephalidae, and Polychrotidae, respectively). Thus far, Dactyloidae and Leiocephalidae have never been described from Europe, but Smith et al.^77^ ascribed an unnamed new species from Messel to the total clade of *Polychrus*. In contrast, the Eocene species *Geiseltaliellus maarius* Smith^23^, from Messel (Fig. 3f) and *Geiseltaliellus longicaudus* Kuhn^78^, from Geiseltal seem to be related to Corytophanidae^23,79^.

**Fig. 4:**
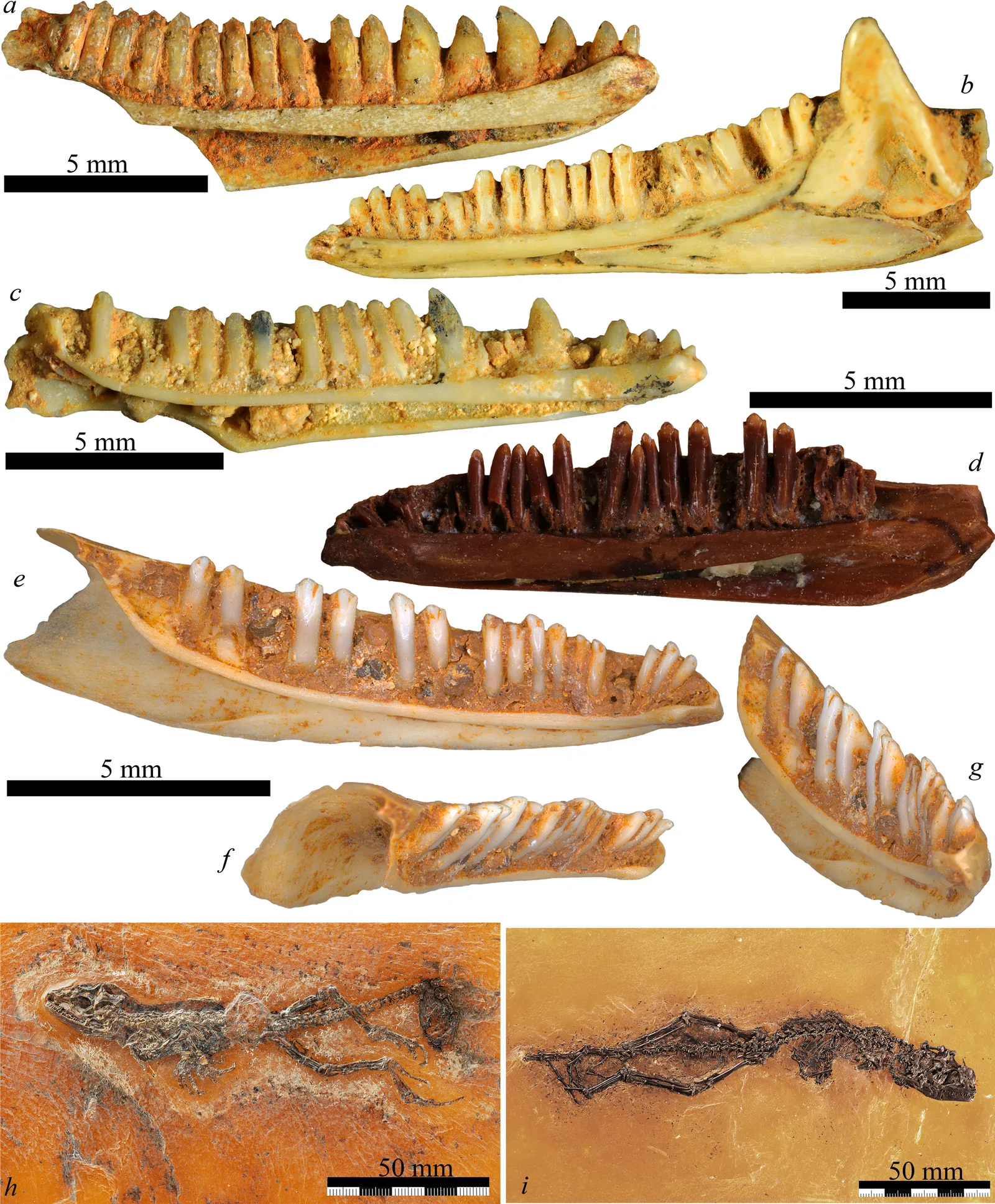
Selected representative specimens of Eocene European Pleurodonta. **a**) Left dentary (MNHN.F.QU17159) of *Pseudolacerta mucronata* from the Eocene of the Phosphorites du Quercy (imprecisely known locality), France, in medial view; **b**) holotype right dentary (MNHN.F.QU17739) of ‘*Pseudolacerta*’ *lamandini* from the Eocene of the Phosphorites du Quercy (imprecisely known locality), France, in medial view; **c**) holotype left dentary (MNHN.F.QU17752) of *Pseudolacerta quercyini* from the Eocene of the Phosphorites du Quercy (imprecisely known locality), France, in medial view; **d**) holotype right dentary (MNHN.F.GRI17405) of *Geiseltaliellus grisolli* from the middle Eocene (MP 16) of Grisolles, Paris Basin, France, in medial view; **e–g**) holotype left dentary (UM BFI 3057) of *Phosphoriguana peritechne* from the late Eocene (MP 17) of La Bouffie, Phosphorites du Quercy, France, in dorsomedial (**e**), posterodorsomedial (**f**), and anterodorsomedial (**g**) views; **h**) paratype skeleton (SMF-ME 2A) of *Geiseltaliellus maarius* from the early–middle Eocene (MP 11) of Messel, Germany; **i**) paratype skeleton (SMF-ME 1769) of *Geiseltaliellus maarius* from the early–middle Eocene (MP 11) of Messel, Germany. Collections of Muséum national d’Histoire naturelle, Paris (**a–d**), Institut des Sciences de l’Évolution, Université de Montpellier, Montpellier (**e–g**), and Messel vertebrate collection, Senckenberg Research Institute and Natural History Museum, Frankfurt am Main (**h–i**). Photographs made by: G.L.G (**a–g**) and Anika Vogel (SMF) (**h–i**).

Among the remaining extinct genera of Pleurodonta from Europe, EOC-UCF-15 differs from *Bifurcodentodon* Čerňanský, Smith, Smith and Folie^52^, from the earliest Eocene of Belgium in lacking the complex tooth morphology of the latter taxon, in which the central cusp is bifurcated, markedly split into two distinct and well-separated portions^52^. It further differs from *Geiseltaliellus* Kuhn^78^, from the early–late Eocene of Germany, France, Belgium, Spain, and Portugal, in possessing more prominent central and accessory cusps, a groove on the apical edge of the central cusp, and generally more robust teeth^23,62,64,80–82^ (see Figs. 3f, 4d). It further differs from *Phosphoriguana* Lemierre & Georgalis^55^ from the late Eocene of France, in having much more robust teeth and with different shape of the central and accessory cusps^55^ (see Fig. 4e–g). It further differs from *Pseudolacerta* De Stefano^83^, from the middle–late Eocene of France in its tricuspid teeth being more robust and with more prominent central and accessory cusps^55,62^ (see Fig. 4a–c).

## Discussion

Pleurodontans in Europe achieved a considerable diversity in the Eocene up to the late Oligocene^23,52–55,75,80–82,84,85^ (see Fig. 4), including beautifully preserved and articulated skeletons from the *Fossillagerstätten* localities of Messel and Geiseltal^23,78,79,86–88^ (see Fig. 4h–i). Pleurodontan fossils in Europe have been known since the second half of the nineteenth century^83,89–91^, though they have been subject to a fascinating and perplexing history of taxonomic identifications, often being misidentified as other lizard groups (see detailed review in^69^). They are entirely absent from Europe in the Paleocene, first appearing there during the earliest Eocene (MP 7); this absence implies that they only dispersed into Europe around the Paleocene–Eocene Thermal Maximum (PETM), together with a number of other immigrants, mainly from North America (see^92^). However, the scarcity of European Paleocene localities that yielded herpetofaunal remains hinders any definite conclusions on the exact timing of their appearance in the continent. Few remains of pleurodontans (or stem pleurodontans) are also known from the Late Cretaceous of Europe (e.g.,^25^), however, these pertain to different lineages than those known from the Paleogene, suggesting that the former forms apparently did not survive the K/Pg extinction event. Although they reached their highest diversity and abundance in Europe in the Eocene (e.g.,^23,52,54,62,64^), pleurodontans survived the “Grande Coupure” and seem to have become locally extirpated at around the late Oligocene^84,85^.

In contrast, the coeval Asian fossil record of pleurodontan iguanians is notoriously poor. Indeed, although the group has representatives in the Cretaceous of the continent, there is no unambiguous fossil occurrence in younger, Cenozoic, localities^8,17^. Fossil taxa from the Paleocene of Mongolia that have been referred or at least considered close to pleurodontans (e.g.,^21,22,24,93,94^) have alternatively been interpreted as being more distantly related^23,95^ or even being of imprecise taxonomic affinities; there is thus no indication that pleurodontan iguanians survived the K/Pg extinction event in Asia^7^.

With regards to Africa, pleurodontans are absent from the extant herpetofauna of the continent, with the notable exception of Opluridae, which are endemic to the islands of Madagascar and Comoros (suggested, among others, to have arrived via a potential dispersal over Antarctica^7^). Pleurodontans are also notoriously absent from the African fossil record, apart from a few Quaternary remains of oplurids from Madagascar^60^ and the Aldabra Atoll^96^. Pleurodontans are also present on a few remote islands in Oceania^1,5,97,98^ and Quaternary fossil remains indicate a higher (than today) insular diversity in this region (e.g., ^4^). Their highest diversity and abundance, on the other hand, is observed in the Americas, where the group is a ubiquitous element across faunas in North, Central, and South America, plus islands in the Caribbean and the Galapagos archipelago^9,10,18–20^. The Americas also host their most abundant fossil record, with several taxa having been described, particularly from the Eocene of North America^13,59,60,76,99–105^.

Squamates are well known to be capable of overwater dispersal, with several lizard and snake groups known to have reached distant landmasses, via rafting on “floating islands of vegetation” (also known in the literature as “floatons”, “flotsams”, “floatsomes”, “floating debris”, or “natural rafts”) (e.g., ^1–8,106–110^). Pleurodontans are excellent at long distance, transoceanic dispersal, as is aptly demonstrated by their highly disjunct modern geographic distribution, which even encompasses some remote oceanic islands^1,5,7,8,97,98,106,107,111,112^. Notably, such extreme cases include the most spectacular example of long-distance oceanic dispersal in the history of terrestrial vertebrates: the iconic extant pleurodontan *Brachylophus* from Fiji, the ancestors of which are thought to have rafted more than 8000 km from North America during the late Paleogene^8^. Such high marine dispersal capabilities of pleurodontans are not only inferred by their disjunct current and fossil distribution including insular areas in the Caribbean and the Galapagos^113^, but also from several direct observations of floating or even swimming by extant individuals (e.g., ^114,115^), including cases of animals that floated for hundreds of kilometers and successfully colonized islands^2^. Dispersals made by eggs (and not directly the animals themselves) have also been proposed, as certain pleurodontan taxa are characterized by a very long egg incubation (e.g., more than nine months in *Brachylophus*) (see ^1,5^). It is also characteristic that the extant diversity of pleurodontans includes a plethora of insular endemics, apparently reflecting the amazing overseas dispersal capabilities of the group^1,5,7–9,19,111^. Indeed, abundant extant insular endemic species of *Leiocephalus*, *Anolis*, *Iguana*, and *Cyclura* inhabit different Caribbean Islands and different species of tropidurids occur in the Galapagos Islands^9,10,18,19,107,111^, while fossil and subfossil remains from a number of islands also attest to the presence of additional, now extinct, insular endemic species (e.g.,^4,116–118^).

The identification of the new fossil material from the middle Eocene (44–43 Ma) Uzunçarşıdere Formation in Anatolia further indicates another successful overseas dispersal for pleurodontans: the colonization of insular Balkanatolia. The insular fauna of Balkanatolia presents a mosaic of taxa of European, Asian, and African affinities^26,27,32,38^, which potentially originated via sweepstakes overseas dispersals^28,29,31^. Balkanatolia remained isolated and insular until around the late Bartonian, at which time it became connected to Asia^30,34^. In particular, the Uzunçarşıdere Formation is notable for its highly endemic mammalian fauna, with a unique composition of traditionally European, Asian, and African clades and evidence of several insular radiations^26–30,32–35^. In addition, several placental mammal clades, namely Rodentia, Carnivora, Hyaenodonta, Perissodactyla, and Artiodactyla are entirely absent in the Orhaniye Basin (the depositional basin in which the Uzunçarşıdere Formation occurs), despite being dominant and diverse components in contemporary faunal assemblages elsewhere across Laurasia, further underscoring the unique insular character of the assemblage.

**Fig. 5:**
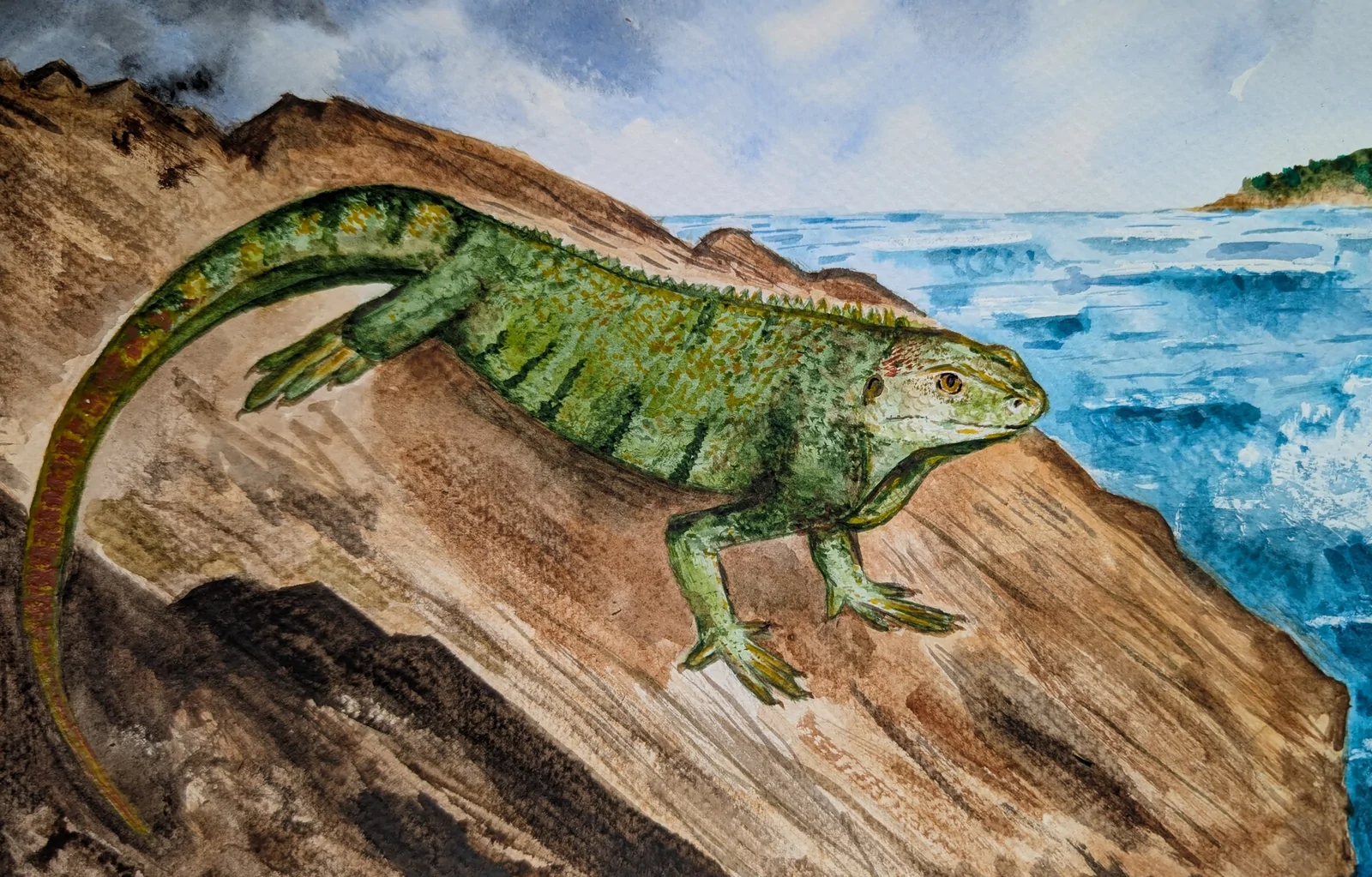
Hypothetical life reconstruction of the ancestor of the new Anatolian pleurodontan. The lizard is envisaged here as standing at the edge of a tree trunk, part of a larger “floating island of vegetation” that is about to reach Balkanatolia. Artwork by Olha Timofieieva.

Insofar as pleurodontan iguanians probably originated in the New World^16^ and probably only dispersed to Europe in the earliest Eocene (e.g.,^52,104^), it is evident that EOC-UCF-15 pertains to a so far unknown taxon which is the product of a dispersal event to Balkanatolia. The total absence of pleurodontans from the Paleogene of Asia and Africa renders Europe as the most likely source of origin for the Anatolian iguanian. It is reasonable to conclude that the ancestor of the Eocene Anatolian pleurodontan reached Balkanatolia via overwater dispersal (Fig. 5). Nevertheless, the incompleteness of the new Anatolian material hinders a more precise identification, and hence a more precise analysis of the biogeographic history of the lineage. The timing of this colonization remains poorly constrained, and we cannot infer precisely when this overseas dispersal event occurred, because the material from the Uzunçarşıdere Formation represents the only evidence substantiating the presence of pleurodontan iguanians in the Balkanatolian Archipelago. Taking into consideration, however, that pleurodontans first appeared in Europe in the earliest Eocene (MP 7), it is reasonable to assume that their dispersal to Anatolia could have occurred between the early and early middle Eocene. During the entire Eocene, including the interval of 44–43 Ma during which this new pleurodontan inhabited Balkanatolia, the distance separating Western Europe from the Balkanatolian archipelago remained relatively limited, on the order of 100 km^39,40^.

The Eocene was a time of intense dispersals for reptiles in the Old World, as is attested by numerous examples between Eurasia and Afro-Arabia, including also overwater dispersals (see discussion in^119^). However, there are no previous data concerning dispersal of reptiles to the isolated faunal province of Balkanatolia. As a matter of fact, Eocene herpetofaunas of Balkanatolia have been previously unknown: although a number of Oligo-Miocene reptiles are known from Anatolia^120–130^, these all originate from much younger localities, at a time when this terrain was already connected to the Asian continent. It is therefore totally unknown whether reptiles dispersed to Balkanatolia in a fashion resembling that of mammals and also which reptile groups, if any, experienced endemic radiations there. Accordingly, despite its fragmentary condition, the new pleurodontan iguanian from the Uzunçarşıdere Formation provides a valuable glimpse into the herpetofaunas of a “Lost World”, the early Paleogene biogeographic province of Balkanatolia.

## Data Availability

All data generated or analysed during this study are included in this published article.
